# Efficacy and safety of intradialytic parenteral nutrition using ENEFLUID_®_ in malnourished patients receiving maintenance hemodialysis: An exploratory, multicenter, randomized, open-label study

**DOI:** 10.1371/journal.pone.0311671

**Published:** 2024-12-12

**Authors:** Hideyuki Kabasawa, Michihiro Hosojima, Eiichiro Kanda, Miho Nagai, Toshiko Murayama, Miyuki Tani, Satoru Kamoshita, Akiyoshi Kuroda, Yoshihiko Kanno

**Affiliations:** 1 Department of Clinical Nutrition Science, Kidney Research Center, Niigata University Graduate School of Medical and Dental Sciences, Niigata City, Niigata, Japan; 2 Department of Health Data Science, Kawasaki Medical School, Kurashiki, Okayama, Japan; 3 Department of Nephrology, Tokyo Medical University, Shinjuku, Tokyo, Japan; 4 Faculty of Human Life Studies, Department of Health and Nutrition, University of Niigata Prefecture, Niigata City, Niigata, Japan; 5 Medical Affairs Department, Research and Development Center, Otsuka Pharmaceutical Factory, Inc. 4F, Chiyoda, Tokyo, Japan; 6 Research and Development Center, Otsuka Pharmaceutical Factory, Inc. 4F, Chiyoda, Tokyo, Japan; University of Otago, NEW ZEALAND

## Abstract

The objective of this study was to investigate the efficacy and safety of intradialytic parenteral nutrition (IDPN) using ENEFLUID_®_ (310 kcal, 550 mL) in mild-moderate malnutrition patients receiving maintenance hemodialysis. A total of 40 adult patients with a Nutritional Risk Index-Japanese Hemodialysis (NRI-JH) score of 5–10 were enrolled in this multicenter, randomized, open-label study. Patients in the intervention group received IDPN using ENEFLUID_®_ via the dialysis circuit 3 times a week for 12 weeks; those in the control group did not. The primary endpoint was change in serum transthyretin (TTR). The secondary endpoints were changes in nutritional laboratory tests, nutritional parameters, food intake, plasma amino acids, and blood glucose. For both groups, mean age (72.1±11.4 years) and BMI (20.3±3.0), and median NRI-JH score [7.0 (interquartile range, 6–8)], did not differ. One patient withdrew before intervention, leaving 20 intervention and 19 control patients. Mean (95% confidence interval) change in serum TTR (mg/dL) at 12 weeks did not differ between groups: Intervention, 1.0 (-1.1–3.2); Control, -0.3 (-2.4–1.9); Intragroup difference, 1.3 (-1.7–4.3); P = 0.41. The values reflecting protein intake at 12 weeks compared to those on the study initiation day increased in the intervention group [the changes of blood urea nitrogen, 9.4 (2.6–16.2) mg/dL; P = 0.007, and normalized protein catabolic rate, 0.10 (0.02–0.18) g/kg/day; P = 0.02]. Mean food protein intake (g/kg/day) at 12 weeks increased in the intervention group and decreased in the control group, and differed between groups: Intervention, 0.12 (-0.03–0.28); Control, -0.18 (-0.43–0.08); Inter-group difference, 0.30 (0.00–0.60); P = 0.050. No adverse events occurred. In patients with mild to moderate malnutrition receiving ENEFLUID_®_ for 12 weeks as IDPN, serum TTR was not improved, decreases in protein intake was mitigated, no adverse events occurred.

**Trial registration** Name of the registry: Japan Registry of Clinical Trials Registration number: jRCTs031220296.

## Introduction

Dialysis patients are getting older as a result of the prolonged survival period due to improvements in dialysis therapy and an increase in the number of the elderly who start to receive dialysis [1,2]. This causes significant problems including sarcopenia, frailty, and protein energy wasting (PEW) which are common malnutrition in the patients with chronic kidney disease (CKD) [3,4]. In patients with CKD receiving dialysis, the intake of food required to address sarcopenia, frailty, and PEW is often insufficient, primarily because of the anorexia that results from uremia, constipation, and polypharmacy [5–7]. Thus, nutritional intervention for malnourished patients who are elderly and on dialysis has become an increasingly urgent issue, particularly in developed countries where the population is aging.

For malnourished patients receiving hemodialysis, the first step in nutritional intervention is counseling, the goal of which is to increase food intake and, if needed, to add oral nutritional supplements (ONS). If this intervention does not result in improved nutritional status, intradialytic parenteral nutrition (IDPN) is recommended as the next step [8–10]. IDPN most often consists of a parenteral nutritional solution containing amino acids, glucose, and lipid which is administered from the venous side of the dialysis circuit. The addition of IDPN has resulted in improved nutritional status and clinical outcomes in patients on hemodialysis in some studies [11–13], but not in others [10]. At the present time, no consensus exists about the patients expected to benefit most from IDPN, the optimal duration that patients should receive IDPN, or the ideal composition of the solution used for IDPN. In order to use IDPN most effectively as a nutritional intervention in malnourished patients receiving dialysis, appropriate indications and methods need to be established.

In 2021, we published the results of a multicenter, prospective, observational study of the efficacy and safety of a 12-week course of IDPN using ENEFLUID_®_ (310 kcal, 550 mL), a solution containing amino acids, glucose, lipid, electrolytes, and water-soluble vitamins [14]. In that study, we evaluated patients whose malnutrition placed them at high risk (≥ 11 points) according to the Nutritional Risk Index-Japanese Hemodialysis (NRI-JH), a nutritional index used for predicting mortality in patients undergoing hemodialysis [15]. Although IDPN was implemented without problems in these high-risk patients, their nutritional parameters did not improve.

The goal of the present exploratory study was to build upon the previous investigation, performing a multicenter, randomized, prospective study of the efficacy (using nutritional parameters, food intake, plasma amino acid concentrations, and blood glucose levels) and safety of IDPN using ENEFLUID_®_ in patients receiving maintenance hemodialysis who have mild to moderate malnutrition (NRI-JH scores of 5 to 10).

## Materials and methods

### Study design and ethics

This was designed as a multicenter, randomized, open-label study. We obtained approval for the study from the Certified Review Board for clinical studies at Tokyo Medical University and registered the study with the Japan Registry of Clinical Trials (jRCTs031220296). The study was conducted in accordance with the Declaration of Helsinki and the Japanese Clinical Trial Act, and written informed consent was obtained from all study patients.

### Study patients

Patients were enrolled in the study from 8 hospitals in Japan from September 7th through December 7th, 2022. Patients included in the study were adults who were receiving maintenance hemodialysis and had mild to moderate malnutrition (scores of 5 to 10) according to the NRI-JH criteria (**S1 Table**) [15]. Because this study focused on malnutrition, serum total cholesterol (T-Cho) of >220mg/dL was not used in the calculation of the NRI-JH score. The following were excluded from eligibility for the study: patients on hemodialysis for less than 6 months; patients for whom ENEFLUID_®_ 550 mL was contraindicated; patients receiving IDPN within 1 month of the study enrollment date; patients who were pregnant or wished to become pregnant; patients with type 1 diabetes mellitus; patients with a severe infectious disease; patients with a diagnosis of or treatment for a malignant tumor within the past 3 years; patients with a history of lower limb amputation.

### Randomization

Study patients were randomly allocated to either the intervention group or the control group in the ratio of 1:1 using the stratified permuted block randomization method (block size, 2 and 4) and using the NRI-JH score (5 to 7 points or 8 to 10 points) as a stratification factor. The randomization was conducted by the Gravity electronic data capture system (Medical Edge Inc.; Tokyo, Japan) which was accessed online.

### Intervention

Patients in the intervention group received ENEFLUID_®_ 550 mL (containing 310 kcal energy, 15 g amino acids, 37.5 g glucose, 10 g lipid, electrolytes, and water-soluble vitamins) (Otsuka Pharmaceutical Factory, Inc.; Tokushima, Japan) (**S2 Table**) from the venous side of the dialysis circuit. This was given over 3 hours or longer and at a constant speed, during each dialysis session, 3 times a week. Patients in the control group did not receive ENEFLUID_®_ 550 mL during dialysis.

The study was initiated on the first dialysis day of the week (Monday or Tuesday) and the study period was 12 weeks. Patients in the study did not receive any other parenteral nutrition solutions (containing amino acids, glucose, or lipid), start to intake any oral nutritional supplements during the study, or have any change in their usual dialysis methods (e.g., dialysates, dialyzer, blood flow rate, and dialysis mode) during the study.

### Outcomes

The primary endpoint used for the study was the change between the study initiation day and the end of the 12-week study in the serum transthyretin (TTR) concentration. The secondary endpoints used for the study were the changes over 12 weeks in nutritional laboratory test results, nutritional parameters, food intake, plasma amino acid concentrations, and blood glucose levels. The examination schedule for evaluating these outcomes is shown in **S1 Fig**.

Blood sampling and the food intake survey were always conducted on the first dialysis day of the week that they were scheduled. Blood samples were obtained from the dialysis circuit before the initiation of dialysis, except the samples for amino acid concentrations, which were obtained both before the initiation and the end of dialysis. Measurements of the concentrations of TTR, retinol binding protein (RBP), transferrin (Tf), T-Cho, magnesium, glycated hemoglobin (HbA1c), glycoalbumin (GA), zinc (Zn), copper (Cu), and amino acids were outsourced to SRL, Inc. (Tokyo, Japan). Other laboratory tests were measured at each hospital.

### Nutritional laboratory tests

Serum concentrations of TTR, RBP, Tf, albumin, cholinesterase, T-Cho, creatinine, and blood urea nitrogen (BUN) were measured on the study initiation day and then 4, 8, and 12 weeks after that. Serum concentrations of Cu and Zn were measured only on the study initiation day and 12 weeks after that.

### Nutritional parameters

Values were obtained for dry weight, body weight, body mass index (BMI), normalized protein catabolic rate (nPCR) [16], NRI-JH [15], Geriatric Nutritional Risk Index (GNRI) [17], and Survival Index (SI) [18] on the study initiation day and 12 weeks after that. The body weight was measured after the dialysis on the final dialysis day (Friday or Saturday) at the previous week of each evaluation point, and BMI, NRI-JH and SI were calculated using the body weight.

### Food intake

Values were obtained for one-day intakes of energy, protein, animal protein, and plant protein per kg of body weight on the study initiation day and 12 weeks after that. This data was acquired using the brief-type self-administered diet history questionnaire (BDHQ) [19], with the questions answered by each patient, with the support of a dietitian. The statistical analysis of the BDHQ results was outsourced to the DHQ Support Center of Gender Medical Research (Tokyo, Japan). Values per body weight (kg) were calculated using the weight corresponds to BMI of 22: 22 x (height [m])^2^ [20].

### Plasma amino acid concentrations

Plasma concentrations of total amino acids (TAA), non-essential amino acids (NEAA), and essential amino acids (EAA) were obtained on the study initiation day and 12 weeks after that. On each of these 2 test days, blood was sampled for plasma amino acid concentrations both before the initiation and the end of dialysis.

### Blood glucose levels

Blood glucose levels were measured using the Free Style Libre (Abbott Japan, LLC; Tokyo, Japan) flash glucose monitoring (FGM) system. During FGM, the Free Style Libre Pro Sensor was put on the upper arm of the patient and the glucose concentration in interstitial fluid was monitored every 15 minutes. After the completion of FGM, the data on the Sensor was read using the Free Style Libre Pro Reader. Blood glucose level data was not available for all patients, because some patients were seen at hospitals without physicians skilled in the use of FGM and other patients had contraindications to the use of FGM (e.g., heart failure).

For each patient undergoing FGM, evaluations were performed 2 weeks after study initiation and 2 weeks before study completion. During 2-week period at such timepoints, blood glucose data was collected; during dialysis for 4 hours, on the day of dialysis for 24 hours, and on the day with no dialysis for 24 hours. FGM results for each patient were used to calculate their individual blood glucose concentration means, durations and frequencies of hypoglycemia (blood glucose < 70 mg/dL) and hyperglycemia (blood glucose ≥ 180 mg/dL) per 24 hours, area over the curve for glucose < 70 mg/dL (AOC_G<70_) per 24 hours_,_ area under the curve for glucose ≥ 180 mg/dL (AUC_G≥180_) per 24 hours, and Time in Range (70 to 180 mg/dL) [21].

### Safety

To assess the safety of IDPN, values for the following were obtained on the study initiation day and 12 weeks after that: hemoglobin, hematocrit, prothrombin time (international normalized ratio), total protein, aspartate aminotransferase, alanine aminotransferase, alkaline phosphatase, lactate dehydrogenase, γ-glutamyl transpeptidase, creatine kinase, amylase, glucose, triglyceride, low-density lipoprotein cholesterol, sodium, potassium, calcium, magnesium, phosphorus, iron, unsaturated iron binding capacity, ferritin, C-reactive protein, brain natriuretic peptide (BNP), HbA1c, and glycoalbumin. Also, adverse events were logged from the day of study initiation to the day of study completion, and these were coded using the Common Terminology Criteria for Adverse Events Version 5.0 (CTCAE v5.0).

### Sample size

This is an exploratory study. The data needed to calculate optimal sample size were not available; therefore, we determined the sample size of 40 patients based on what would be practical to achieve at each site.

### Statistical analysis

The following study patients were excluded from those whose data was subjected to statistical analysis: patients who had no evaluation data; patients who withdrew consent; and intervention group patients who did not receive ENEFLUID_®_.

Patient characteristics and other categorical variables such as dialysis conditions were described using frequencies and proportions. Continuous variables were summarized using means and standard deviations (SDs) or medians and interquartile ranges (IQRs). Intergroup bias was assessed using the absolute standardized differences (ASD) [22].

For the items evaluated at 4 timepoints, summary statistics for each item at each timepoint were calculated, after which changes in the value of the item over the timepoints were calculated. These results were compared between the 2 groups using the mixed-effects model for repeated measures (MMRM), where the patient was set as the random effect and the study group, timepoint, and interaction term were set as the fixed effects. The restricted maximum likelihood method was used as the estimation method. A first-order autoregressive structure for covariance was used. Within the mixed-effects model, the least squares (LS) means and 95% confidence intervals (95% CI) of the changes from each timepoint to the 12-week timepoint were calculated. As MMRM can provide estimates close to the true values even when data are missing, the analysis was performed using all patients’ data including drop-out patients’ ones.

For the items evaluated at 2 timepoints, summary statistics for each item and changes in the values of the items between the timepoints were calculated, with results reported as means and 95% CI. The independent t-test was used to compare the changes between the 2 groups. The paired-samples t-test was used to compare the results before study initiation and at 12 weeks within each group.

For food intake results, a statistical analysis was performed using a patient group where the patients whose energy intake was less than a quarter or 2 times or more of the recommended amount were additionally excluded due to measurements being suspicious. For FGM results, blood glucose concentration means for each group were expressed as means ± SDs; frequencies and durations of hypoglycemia and hyperglycemia were expressed as medians and IQRs of occurrences per 24 hours and hours per 24 hours, respectively; and AOC_G<70_ per 24 hours, AUC_G≥180_ per 24 hours, and Time in Range were also each expressed as medians and IQRs. The independent t-test or the Mann-Whitney U test were used for comparisons between the 2 groups.

For adverse events, the number of patients who developed events, number of events, and event occurrence percentages were recorded. Statistical significance was defined at the two-sided 5% (P < 0.05) level. SPSS version 24.0 (IBM Japan, Ltd) was used for all statistical analyses.

## Results

**Fig 1:**
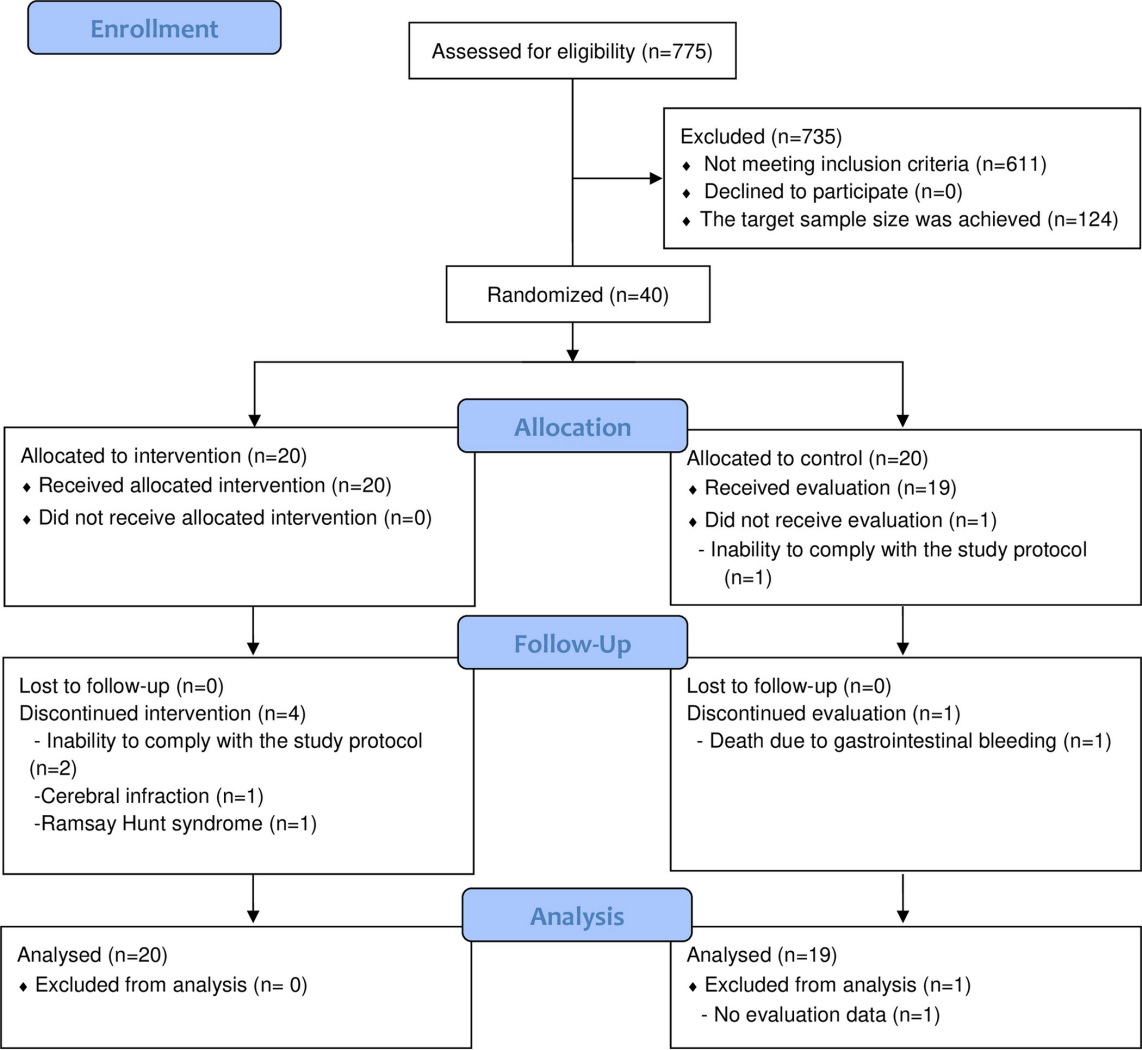
Flowchart of study in which 40 patients were enrolled and then randomized to the intervention (12-week course of intradialytic parenteral nutrition [IDPN] using ENEFLUID_®_) or control groups in a ratio of 1:1 using the stratified permuted block randomization method. A total of 6 of those patients were withdrawn from the study at various timepoints over the 12 weeks for reasons as noted.

Among 775 patients screened for eligibility, 611 patients did not meet NRI-JH criteria; consent was obtained from 40 of the 164 who met the eligibility criteria. Of the 164 patients who met the criteria, 124 patients did not receive an explanation of the study because the target sample size was achieved. A total of 40 patients were initially enrolled in the study and randomly allocated to either the intervention group or the control group in the ratio of 1:1 (**Fig 1**). Among those, 6 patients were withdrawn from the study at various timepoints, because of inability to comply with the protocol (n = 3), cerebral infarction (n = 1), Ramsay Hunt syndrome (n = 1) or death due to gastrointestinal bleeding (n = 1). This ultimately resulted in a total of 34 patients (16 patients in the intervention group and 18 patients in the control group) who completed the study.

However, 39 patients (excluding a single patient who was withdrawn before the study initiation day and so had no evaluation data) were included in many of the statistical analyses, including those involving MMRM, regardless of whether they completed the study or not. The demographic and clinical characteristics of the study patients at the beginning of the study and the dialysis conditions, access routes, and modes are shown in **Table 1**.

**Table 1:** Demographic and clinical characteristics of 39 adult patients with malnutrition on maintenance hemodialysis, by those receiving vs. not receiving intradialytic parenteral nutrition (IDPN) beginning September through December 2022.

*Characteristics*		Intervention (IDPN) group	Control group	ASD
		n = 20	n = 19	
**Age**, mean±SD, *years*		72.1 ± 8.6	72.1 ± 14	0.00
**Height**, mean±SD, *cm*		159.5 ± 11.0	162.8 ± 10.5	0.30
**Body weight**, mean±SD, *kg*		51.3 ± 9.5	55.0 ± 12.5	0.33
**BMI**, mean±SD, *kg/m*^*2*^		20.0 ± 2.5	20.6 ± 3.5	0.19
**Sex**, n (%)	***Male***	12 (60.0)	15 (78.9)	0.42
	***Female***	8 (40.0)	4 (21.1)	
**Primary** **diseases**, n (%)	***Diabetic nephropathy***	9 (45.0)	7 (36.8)	0.16
	***Chronic glomerulonephritis***	3 (15.0)	5 (26.3)	0.28
	***Nephrosclerosis***	0 (0.0)	3 (15.8)	0.61
	***Polycystic kidney disease***	2 (10.0)	1 (5.3)	0.18
	***Other***	7 (35.0)	3 (15.8)	0.45
**NRI-JH**, median [IQR]		7.0 [5.0, 8.0]	7.0 [5.0, 8.0]	-
**Dialysis conditions**	***Blood flow rate***, mean±SD, *mL/min*	211.5 ± 24.6	206.3 ± 23.6	0.22
	***HD duration*,** mean±SD, *min*	240.5 ± 27.5	249.6 ± 28.3	0.33
	***Kt/Vsp*,** mean±SD	1.19 ± 0.27	1.17 ± 0.25	0.08
**Dialysis blood access**, n (%)	***Arteriovenous fistula***	15 (75.0)	15 (78.9)	0.09
	***Arteriovenous graft***	3 (15.0)	0 (0.0)	0.59
	***Subcutaneously fixed superficial artery***	0 (0.0)	2 (10.5)	0.49
	***Long-term indwelling catheter***	2 (10.0)	2 (10.5)	0.02
**Dialysis mode**, n (%)	***HD***	14 (70.0)	16 (84.2)	0.34
	***Pre-dilution online HDF***	6 (30.0)	3 (15.8)	

***Abbreviations***: SD, standard deviation; ASD, absolute standardized difference; BMI, body mass index; NRI-JH, Nutritional Risk Index-Japanese Hemodialysis; HD, hemodialysis; Kt/Vsp, single-pool Kt/V; HDF, hemodiafiltration; IQR, interquartile range.

### Nutritional laboratory tests and parameters

Results for nutritional laboratory tests and nutritional parameters are shown in **Tables 2–4**. The mean (95% CI) change in serum TTR between the study initiation day and 12 weeks was not significant for either group, and the amount of change did not differ significantly between the 2 groups: Intervention group, 1.0 (-1.1–3.2) mg/dL; Control group, -0.3 (-2.4–1.9) mg/dL; Inter-group difference, 1.3 (-1.7–4.3) mg/dL; P = 0.41. The BUN, Zn, and nPCR values at 12 weeks compared to those on the study initiation day increased significantly in the intervention group [the changes of BUN, 9.4 (2.6–16.2) mg/dL; P = 0.007, Zn, 10 (2–18) μg/dL; P = 0.02 and nPCR, 0.10 (0.02–0.18) g/kg/day; P = 0.02], but did not change significantly in the control group. For all other nutritional laboratory tests and nutritional parameters, no significant changes occurred between the study initiation day and 12 weeks, nor were there significant differences between the 2 groups.

**Table 2:** Nutritional laboratory test results for 39 adult patients with malnutrition on maintenance hemodialysis, measured and compared at 4 timepoints, and compared between those receiving intradialytic parenteral nutrition (IDPN) and controls receiving no intervention, beginning September through December 2022.

	Intervention (IDPN) group		Control group		Difference (Intervention—Control)	
	n = 20		n = 19			
	LS mean (95% CI)	P-value^a^	LS mean (95% CI)	P-value^a^	LS mean (95% CI)	P-value^b^
**Transthyretin**, *mg/dL*						
Study initiation day	21.1 (17.9 to 24.3)	–	21.0 (17.8 to 24.3)	–	–	–
4 weeks	23.0 (19.8 to 26.2)	–	21.7 (18.4 to 25.0)	–	–	–
8 weeks	22.5 (19.2 to 25.7)	–	22.6 (19.3 to 25.9)	–	–	–
12 weeks	22.1 (18.8 to 25.3)	–	20.8 (17.5 to 24.1)	–	–	–
Change at 12 weeks	1.0 (-1.1 to 3.2)	0.34	-0.3 (-2.4 to 1.9)	0.82	1.3 (-1.7 to 4.3)	0.41
**Retinol binding protein**, *mg/dL*						
Study initiation day	6.2 (5.2 to 7.2)	–	6.2 (5.2 to 7.2)	–	–	–
4 weeks	6.6 (5.6 to 7.5)	–	6.5 (5.5 to 7.5)	–	–	–
8 weeks	6.4 (5.4 to 7.4)	–	6.5 (5.5 to 7.5)	–	–	–
12 weeks	6.6 (5.6 to 7.6)	–	6.5 (5.4 to 7.5)	–	–	–
Change at 12 weeks	0.4 (-0.1 to 0.9)	0.13	0.2 (-0.3 to 0.8)	0.35	0.2 (-0.6 to 0.9)	0.67
**Transferrin**, *mg/dL*						
Study initiation day	184 (167 to 202)	–	176 (159 to 194)	–	–	–
4 weeks	191 (174 to 209)	–	182 (164 to 200)	–	–	–
8 weeks	184 (166 to 201)	–	181 (163 to 199)	–	–	–
12 weeks	178 (160 to 196)	–	176 (158 to 194)	–	–	–
Change at 12 weeks	-6 (-18 to 6)	0.32	0 (-12 to 12)	>0.99	-6 (-23 to 11)	0.47
**Albumin**, *g/dL*						
Study initiation day	3.3 (3.1 to 3.5)	–	3.3 (3.2 to 3.5)	–	–	–
4 weeks	3.4 (3.2 to 3.6)	–	3.5 (3.3 to 3.7)	–	–	–
8 weeks	3.3 (3.1 to 3.5)	–	3.5 (3.3 to 3.6)	–	–	–
12 weeks	3.3 (3.1 to 3.5)	–	3.4 (3.2 to 3.6)	–	–	–
Change at 12 weeks	0.0 (-0.1 to 0.2)	0.76	0.1 (-0.1 to 0.2)	0.38	0.0 (-0.3 to 0.1)	0.69
**Cholinesterase**, *U/L*						
Study initiation day	183 (153 to 212)	–	201 (171 to 232)	–	–	–
4 weeks	194 (165 to 224)	–	206 (176 to 236)	–	–	–
8 weeks	182 (153 to 212)	–	205 (175 to 236)	–	–	–
12 weeks	186 (156 to 216)	–	201 (171 to 232)	–	–	–
Change at 12 weeks	3 (-10 to 16)	0.61	0 (-13 to 13)	>0.99	3 (-15 to 22)	0.71
**Total cholesterol**, *mg/dL*						
Study initiation day	143 (126 to 159)	–	125 (108 to 142)	–	–	–
4 weeks	143 (126 to 159)	–	127 (110 to 144)	–	–	–
8 weeks	142 (125 to 159)	–	125 (108 to 142)	–	–	–
12 weeks	139 (123 to 156)	–	125 (108 to 142)	–	–	–
Change at 12 weeks	-4 (-13 to 6)	0.48	0 (-10 to 10)	>0.99	-4.0 (-17 to 10)	0.62
**Creatinine**, *mg/dL*						
Study initiation day	8.0 (7.1 to 8.8)	–	8.5 (7.6 to 9.3)	–	–	–
4 weeks	7.7 (6.9 to 8.6)	–	8.2 (7.3 to 9.0)	–	–	–
8 weeks	8.2 (7.4 to 9.1)	–	8.1 (7.3 to 9.0)	–	–	–
12 weeks	8.5 (7.6 to 9.3)	–	8.2 (7.4 to 9.0)	–	–	–
Change at 12 weeks	0.5 (-0.3 to 1.4)	0.23	-0.3 (-1.1 to 0.6)	0.56	0.8 (-0.4 to 2.0)	0.21
**Blood urea nitrogen**, *mg/dL*						
Study initiation day	52.8 (45.7 to 60.0)	–	53.1 (45.9 to 60.3)	–	–	–
4 weeks	57.3 (50.3 to 64.3)	–	52.0 (44.9 to 59.2)	–	–	–
8 weeks	57.9 (50.9 to 65.0)	–	57.4 (50.1 to 64.6)	–	–	–
12 weeks	62.2 (54.9 to 69.5)	–	55.3 (48.0 to 62.6)	–	–	–
Change at 12 weeks	9.4 (2.6 to 16.2)	**0.007**	2.2 (-4.5 to 9.0)	0.51	7.2 (-2.4 to 16.8)	0.14

^a^ 12 weeks vs. study initiation day, P-values based on mixed-effects model for repeated measures, and a first-order autoregressive covariance structure was used.

^b^ Intervention group vs Control group, P-values based on mixed-effects model for repeated measures, and a first-order autoregressive covariance structure was used.

***Abbreviations***: LS, least squares; CI, confidence interval.

**Table 3:** Nutritional laboratory test results for 34 adult patients with malnutrition on maintenance hemodialysis, measured and compared at 2 timepoints, and compared between those receiving intradialytic parenteral nutrition (IDPN) and controls receiving no intervention, beginning September through December 2022.

	Intervention (IDPN) Group			Control Group			Difference (Intervention—Control)	
	n	mean (95% CI)	P-value^a^	n	mean (95% CI)	P-value^a^	mean (95% CI)	P-value^b^
**Copper**, *μg/dL*								
Study initiation day	15	87 (62 to 111)	–	18	86 (75 to 97)	–	–	–
12 weeks		87 (62 to 113)	–		90 (79 to 101)	–	–	–
Change at 12 weeks		1 (-7 to 8)	0.82		4 (-3 to 11)	0.22	-3 (-13 to 6)	0.50
**Zinc**, *μg/dL*								
Study initiation day	16	59 (52 to 66)	–	18	59 (54 to 64)	–	–	–
12 weeks		69 (58 to 80)	–		62 (57 to 67)	–	–	–
Change at 12 weeks		10 (2 to 18)	**0.02**		3 (-2 to 8)	0.19	7 (-2 to 16)	0.14

^a^ 12 weeks vs. study initiation day, P-values based on paired-samples t-test.

^b^ Intervention group vs Control group, P-values based on unpaired t-test.

**Abbreviations:** CI, confidence interval.

**Table 4:** Nutritional parameter results for 34 adult patients with malnutrition on maintenance hemodialysis, measured and compared at 2 timepoints, and compared between those receiving intradialytic parenteral nutrition (IDPN) and controls receiving no intervention, beginning September through December 2022.

	Intervention (IDPN) group			Control group			Difference (Intervention—Control)	
	n	mean (95% CI)	P-value^a^	n	mean (95% CI)	P-value^a^	mean (95% CI)	P-value^b^
**Dry weight**, *kg*								
Study initiation day	16	52.8 (48.6 to 57.1)	–	18	55.9 (49.7 to 62.1)	–	–	–
12 weeks		53.0 (48.8 to 57.2)	–		55.8 (49.4 to 62.2)	–	–	–
Change at 12 weeks		0.2 (-0.3 to 0.6)	0.43		-0.1 (-0.4 to 0.3)	0.63	0.3 (-0.3 to 0.8)	0.35
**Body weight**, *kg*								
Study initiation day	16	52.9 (48.7 to 57.1)	–	18	56.0 (49.8 to 62.2)	–	–	–
12 weeks		53.0 (48.8 to 57.2)	–		55.9 (49.5 to 62.3)	–	–	–
Change at 12 weeks		0.1 (-0.4 to 0.6)	0.61		-0.1 (-0.6 to 0.3)	0.54	0.3 (-0.4 to 0.9)	0.43
**Body mass index**								
Study initiation day	16	20.4 (19.2 to 21.7)	–	18	20.6 (18.8 to 22.4)	–	–	–
12 weeks		20.5 (19.2 to 21.8)	–		20.5 (18.7 to 22.4)	–	–	–
Change at 12 weeks		0.1 (-0.1 to 0.3)	0.50		-0.1 (-0.2 to 0.1)	0.52	0.1 (-0.1 to 0.4)	0.35
**Normalized protein catabolic rate**, *g/kg/day*								
Study initiation day	13	0.65 (0.56 to 0.75)	–	16	0.67 (0.61 to 0.73)	–	–	–
12 weeks		0.76 (0.66 to 0.85)	–		0.69 (0.62 to 0.77)	–	–	–
Change at 12 weeks		0.10 (0.02 to 0.18)	**0.02**		0.02 (-0.03 to 0.07)	0.36	0.08 (-0.01 to 0.17)	0.07
**Nutritional risk index-Japanese hemodialysis**								
Study initiation day	16	6.4 (5.0 to 7.7)	–	18	6.3 (5.5 to 7.1)	–	–	–
12 weeks		5.8 (4.7 to 7.0)	–		6.8 (6.1 to 7.5)	–	–	–
Change at 12 weeks		-0.6 (-2.0 to 0.8)	0.40		0.5 (-0.5 to 1.5)	0.31	-1.1 (-2.7 to 0.6)	0.19
**Geriatric nutritional risk index**								
Study initiation day	16	87.5 (84.0 to 90.9)	–	18	88.0 (85.1 to 91.0)	–	–	–
12 weeks		88.7 (85.2 to 92.1)	–		89.0 (86.1 to 91.9)	–	–	–
Change at 12 weeks		1.2 (-1.0 to 3.4)	0.26		1.0 (-0.8 to 2.7)	0.26	0.3 (-2.4 to 2.9)	0.85
**Survival index**								
Study initiation day	16	13.8 (10.9 to 16.6)	–	18	15.4 (11.5 to 19.3)	–	–	–
12 weeks		14.1 (11.0 to 17.3)	–		15.8 (11.8 to 19.7)	–	–	–
Change at 12 weeks		0.4 (-0.8 to 1.6)	0.48		0.3 (-0.6 to 1.2)	0.43	0.1 (-1.3 to 1.4)	0.94

^a^ 12 weeks vs. study initiation day, P-values based on paired-samples t-test.

^b^ Intervention group vs Control group, P-values based on unpaired t-test.

***Abbreviation*:** CI, confidence interval.

### Food intake

Results for food intake are shown in **Tables 5**and **S3**. The mean (95% CI) energy intake at 12 weeks increased in the intervention group, though not significantly, it decreased significantly in the control group, and it differed significantly between the 2 groups: Intervention group, 1.8 (-2.4–6.0) kcal/kg/day; Control group, -4.0 (-8.0 –-0.1) kcal/kg/day; Inter-group difference, 5.9 (0.3–11.4) kcal/kg/day; P = 0.04. Protein intake differed between the 2 groups: Inter-group difference, 0.30 (0.00–0.60) g/kg/day; P = 0.050]. When the same analyses were repeated using all patients’ data, the results were similar (**S3 Table**).

**Table 5:** Food intake^a^ results for 31 adult patients^b^ with malnutrition on maintenance hemodialysis, measured and compared at 2 timepoints, and compared between those receiving intradialytic parenteral nutrition (IDPN) and controls receiving no intervention, beginning September through December 2022.

	Intervention (IDPN) group		Control group		Difference (Intervention—Control)	
	n = 14		n = 17			
	mean (95% CI)	P-value^c^	mean (95% CI)	P-value^c^	mean (95% CI)	P-value^d^
**Energy intake**, *kcal/kg/day*						
Study initiation day	24.5 (20.0 to 29.0)	–	30.5 (26.6 to 34.3)	–	–	–
12 weeks	26.3 (21.5 to 31.1)	–	26.4 (21.9 to 31.0)	–	–	–
Change at 12 weeks	1.8 (-2.4 to 6.0)	0.36	-4.0 (-8.0 to -0.1)	**0.045**	5.9 (0.3 to 11.4)	**0.04**
**Protein intake**, *g/kg/day*						
Study initiation day	0.89 (0.64 to 1.13)	–	1.29 (1.07 to 1.51)	–	–	–
12 weeks	1.01 (0.73 to 1.29)	–	1.11 (0.86 to 1.36)	–	–	–
Change at 12 weeks	0.12 (-0.03 to 0.28)	0.11	-0.18 (-0.43 to 0.08)	0.16	0.30 (0.00 to 0.60)	0.050
**Animal protein intake**, *g/kg/day*						
Study initiation day	0.51 (0.33 to 0.70)	–	0.79 (0.59 to 0.99)	–	–	–
12 weeks	0.60 (0.40 to 0.80)	–	0.68 (0.49 to 0.87)	–	–	–
Change at 12 weeks	0.12 (-0.04 to 0.21)	0.16	-0.11 (-0.35 to 0.12)	0.33	0.20 (-0.08 to 0.47)	0.15
**Plant protein intake**, *g/kg/day*						
Study initiation day	0.37 (0.30 to 0.44)	–	0.50 (0.41 to 0.58)	–	–	–
12 weeks	0.41 (0.33 to 0.49)	–	0.43 (0.36 to 0.51)	–	–	–
Change at 12 weeks	0.04 (-0.02 to 0.10)	0.14	-0.06 (-0.12 to -0.01)	**0.04**	0.11 (0.03 to 0.18)	**0.01**

^a^ Data obtained using the brief-type self-administered diet history questionnaire (BDHQ) [19].

^b^ The group for this analysis was created by excluding 3 who had energy intake on the study initiation day <1/4 or ≥ 2 times the recommended amount.

^c^ 12 weeks vs. study initiation day, P-values based on paired-samples t-test.

^d^ Intervention group vs Control group, P-values based on unpaired t-test.

***Abbreviation*:** CI, confidence interval.

### Plasma amino acid concentrations

Results for plasma amino acid concentrations are shown in **Table 6**. The pre- to post-dialysis decrease in mean (95% CI) TAA at 12 weeks was smaller in the intervention group than in the control group. The amount of decrease in TAA was significantly different between the 2 groups: Intervention group, -759 (-1191 –-328) nmol/mL; Control group, -1212 (-1401 –-1024) nmol/mL; Inter-group difference, 453 (19–887) nmol/mL; P = 0.04. Also, from pre- to post-dialysis at 12 weeks, the mean (95% CI) EAA increased in the intervention group and decreased in the control group. The difference in the amount of change in EAA between the 2 groups was significant: Intervention group, 89 (-82–260) nmol/mL; Control group, -237 (-285 –-190) nmol/mL; Inter-group difference, 326 (165–488) nmol/mL; P < 0.001.

**Table 6:** Plasma amino acid difference before and after dialysis for 34 adult patients with malnutrition on maintenance hemodialysis, compared between the study initiation day and at 12 weeks, and compared between those receiving intradialytic parenteral nutrition (IDPN) and controls receiving no intervention, beginning September through December 2022.

	Intervention (IDPN) group		Control group		Difference (Intervention—Control)	
	n = 16		n = 18			
	mean (95% CI)	P-value^a^	mean (95% CI)	P-value^a^	mean (95% CI)	P-value^b^
**Total amino acids**, *nmol/mL*						
Study initiation day	-647 (-1201 to -93)	–	-1169 (-1349 to -989)	–	522 (-9 to 1053)	0.054
12 weeks	-759 (-1191 to -328)	0.50	-1212 (-1401 to -1024)	0.71	453 (19 to 887)	**0.04**
**Non-essential amino acids**, *nmol/mL*						
Study initiation day	-867 (-1342 to -392)	–	-925 (-1056 to -793)	–	58 (-390 to 506)	0.79
12 weeks	-848 (-1166 to -530)	0.88	-975 (-1131 to -819)	0.59	127 (-201 to 455)	0.44
**Essential amino acids**, *nmol/mL*						
Study initiation day	220 (99 to 340)	–	-244 (-306 to -182)	–	464 (338 to 590)	**<0.001**
12 weeks	89 (-82 to 260)	0.06	-237 (-285 to -190)	0.82	326 (165 to 488)	**<0.001**

^a^ 12 weeks vs. study initiation day, P-values based on paired-samples t-test.

^b^ Intervention group vs Control group, P-values based on unpaired t-test.

***Abbreviation*:** CI, confidence interval.

### Blood glucose levels

Results for blood glucose levels are shown in **Table 7**. The median (IQR) durations of hypoglycemia during dialysis for 2 weeks after the study initiation and before the study completion were less in the intervention group than in the control group resulting in a significant difference between 2 groups: 2 weeks after study initiation, 0.0 (0.0–0.5) hours in the intervention group and 1.7 (0.0–14.2) hours in the control group; P = 0.01; 2 weeks before study completion, 0.0 (0.0–1.9) hours in the intervention group and 3.0 (0.0–7.6) hours in the control group; P = 0.04. The results of occurrences of hypoglycemia and AOC_G<70_ were similar to the result of duration of hypoglycemia. The duration and occurrences of hyperglycemia and AUC_G≥180_ during dialysis, for 2 weeks after study initiation and before study completion did not differ between both groups.

**Table 7:** Blood glucose concentration results based on flash glucose monitoring (FGM) for adult patients with malnourishment on maintenance hemodialysis, evaluated 2 weeks after study initiation and 2 weeks before study completion, and compared between those receiving intradialytic parenteral nutrition (IDPN) and controls receiving no intervention, beginning September through December 2022.

Evaluation period		Evaluation timepoint	Intervention (IDPN) group	Control group	P-value^a^
			Patients evaluated (n at 2 weeks after study initiation, n at 2 weeks before study completion)		
			(14, 11)	(13, 13)	
**Mean blood glucose**, mean±SD, *mg/dL*					
During dialysis (for 4 hours)		2 weeks after study initiation	107.3 ± 16.8	88.6 ± 25.2	**0.03**
		2 weeks before study completion	112.3 ± 23.8	89.8 ± 23.3	**0.03**
On day of dialysis (for 24 hours)		2 weeks after study initiation	97.1 ± 14.5	104.7 ± 27.8	0.38
		2 weeks before study completion	98.9 ± 17.2	97.8 ± 27.1	0.92
On day with no dialysis (for 24 hours)		2 weeks after study initiation	97.0 ± 20.5	106.4 ± 30.7	0.36
		2 weeks before study completion	95.9 ± 16.3	101.3 ± 37.0	0.66
**Hypoglycemia (glucose < 70 mg/dL)**, median [IQR], *duration (h) per 24h*					
During dialysis (for 4 hours)	2 weeks after study initiation		0.0 [0.0, 0.5]	1.7 [0.0, 14.2]	**0.01**
	2 weeks before study completion		0.0 [0.0, 1.9]	3.0 [0.0, 7.6]	**0.04**
On day of dialysis (for 24 hours)	2 weeks after study initiation		3.7 [1.1, 6.2]	1.9 [0.2, 11.3]	0.83
	2 weeks before study completion		3.7 [2.3, 7.7]	4.3 [1.2 10.6]	0.64
On day with no dialysis (for 24 hours)	2 weeks after study initiation		4.4 [2.2, 6.3]	1.7 [0.1, 8.0]	0.27
	2 weeks before study completion		4.5 [1.7, 7.8]	6.3 [0.8, 11.0]	0.84
**Hypoglycemia (glucose < 70 mg/dL)**, median [IQR], *occurrences per 24h*					
During dialysis (for 4 hours)		2 weeks after study initiation	0.0 [0.0, 2.0]	6.9 [0.0, 56.7]	**0.01**
		2 weeks before study completion	0.0 [0.0, 7.6]	12.0 [0.0, 30.3]	**0.04**
On day of dialysis (for 24 hours)		2 weeks after study initiation	14.7 [4.4, 24.6]	7.6 [0.7, 45.3]	0.83
		2 weeks before study completion	14.6 [9.2, 30.8]	17.0 [4.7, 42.3]	0.64
On day with no dialysis (for 24 hours)		2 weeks after study initiation	17.6 [8.8, 25.2]	6.9 [0.3, 32.0]	0.27
		2 weeks before study completion	17.8 [6.9, 31.1]	25.3 [3.2, 44.1]	0.84
**AOC**_**G<70**_, median [IQR], *mg*∙*min/dL per 24h*					
During dialysis (for 4 hours)		2 weeks after study initiation	0.0 [0.0, 2.9]	5.4 [0.0, 133.9]	**0.02**
		2 weeks before study completion	0.0 [0.0, 8.4]	6.0 [0.0, 45.3]	0.07
On day of dialysis (for 24 hours)		2 weeks after study initiation	25.8 [6.6, 51.9]	9.7 [0.7, 111.4]	0.87
		2 weeks before study completion	20.1 [14.4, 63.6]	19.5 [5.0, 125.5]	1.00
On day with no dialysis (for 24 hours)		2 weeks after study initiation	26.7 [14.7, 61.3]	12.5 [0.1, 83.4]	0.20
		2 weeks before study completion	33.9 [8.7, 72.2]	36.9 [3.5, 134.0]	0.66
**Hyperglycemia (glucose ≥ 180 mg/dL)**, median [IQR], *duration (h) per 24h*					
During dialysis (for 4 hours)	2 weeks after study initiation		0.0 [0.0, 0.0]	0.0 [0.0, 0.0]	0.30
	2 weeks before study completion		0.0 [0.0, 0.0]	0.0 [0.0, 0.0]	0.86
On day of dialysis (for 24 hours)	2 weeks after study initiation		0.1 [0.0, 0.4]	1.0 [0.0, 2.6]	0.09
	2 weeks before study completion		0.1 [0.0, 0.8]	0.2 [0.0, 1.4]	0.47
On day with no dialysis (for 24 hours)	2 weeks after study initiation		0.1 [0.0, 0.8]	0.0 [0.0, 1.1]	0.80
	2 weeks before study completion		0.1 [0.0, 0.6]	0.3 [0.0, 0.8]	0.68
**Hyperglycemia (glucose ≥ 180 mg/dL)**, median [IQR], *occurrences per 24h*					
During dialysis (for 4 hours)		2 weeks after study initiation	0.0 [0.0, 0.0]	0.0 [0.0, 0.0]	0.30
		2 weeks before study completion	0.0 [0.0, 0.0]	0.0 [0.0, 0.0]	0.86
On day of dialysis (for 24 hours)		2 weeks after study initiation	0.4 [0.0, 1.5]	3.8 [0.0, 10.5]	0.09
		2 weeks before study completion	0.4 [0.0, 3.0]	0.8 [0.0, 5.7]	0.47
On day with no dialysis (for 24 hours)		2 weeks after study initiation	0.5 [0.0, 3.2]	0.1 [0.0, 4.5]	0.80
		2 weeks before study completion	0.3 [0.0, 2.4]	1.0 [0.0, 3.4]	0.68
**AUC**_**G≥180**_, median [IQR], *mg*∙*min/dL per 24h*					
During dialysis (for 4 hours)		2 weeks after study initiation	0.0 [0.0, 0.0]	0.0 [0.0, 0.0]	0.30
		2 weeks before study completion	0.0 [0.0, 0.0]	0.0 [0.0, 0.0]	0.86
On day of dialysis (for 24 hours)		2 weeks after study initiation	0.3 [0.0, 6.0]	10.5 [0.0, 75.1]	0.07
		2 weeks before study completion	0.8 [0.0, 5.7]	1.0 [0.0, 25.0]	0.65
On day with no dialysis (for 24 hours)		2 weeks after study initiation	2.2 [0.0, 12.2]	0.3 [0.0, 24.1]	0.80
		2 weeks before study completion	0.2 [0.0, 8.2]	2.9 [0.0, 19.5]	0.63
**Time in range,** median [IQR], *%*					
During dialysis (for 4 hours)		2 weeks after study initiation	100 [98, 100]	86 [41, 99]	**0.005**
		2 weeks before study completion	100 [90, 100]	88 [69, 97]	0.06
On day of dialysis (for 24 hours)		2 weeks after study initiation	84 [74, 94]	77 [51, 94]	0.28
		2 weeks before study completion	83 [68, 89]	79 [51, 88]	0.37
On day with no dialysis (for 24 hours)		2 weeks after study initiation	79 [73, 86]	85 [54, 96]	0.77
		2 weeks before study completion	81 [68, 87]	69 [43, 90]	0.42

^a^ Intervention group vs Control group P-values based on unpaired t-test (for mean) or Mann-Whitney U test (for occurrences and duration of hypoglycemia, hyperglycemia, AOC _G<70_, AUC _G≥180_, and time in range).

**Abbreviations:** SD, standard deviation; AOC _G<70,_ area over the curve for glucose < 70 mg/dL; IQR, interquartile range; AUC _G≥180,_ area under the curve for glucose ≥ 180 mg/dL.

The median (IQR) Time in Range during dialysis, for 2 weeks after study initiation, was 100 (98, 100) % in the intervention group and 86 (41, 99) % in the control group, representing a significant difference between the 2 groups (P = 0.005). The Time in Range during dialysis for 2 weeks before study completion, was 100 (90, 100) % in the intervention group and 88 (69, 97) % in the control group, indicating a similar though not significant difference between the 2 groups (P = 0.06).

### Safety

Results for safety-related clinical laboratory tests are shown in **S4 and S5 Tables**. The mean (95% CI) BNP at 12 weeks increased significantly in the control group, decreased in the intervention group, and the difference between the 2 groups was significant: Control group, 425 (95–754) pg/mL; Intervention group, -159 (-491–173) pg/mL; Inter-group difference, -584 (-1051 –-116) pg/mL; P = 0.02. There were no significant differences in the glycemic control (HbA1c or GA) test results between the 2 groups. No adverse events related to ENEFLUID_®_ were observed during the study period.

## Discussion

In this exploratory study, we investigated the effects of a 12-week course of IDPN using ENEFLUID_®_ on nutritional labs and parameters, food intake, plasma amino acids, and blood glucose in patients receiving maintenance hemodialysis and with mild to moderate malnutrition according to NRI-JH criteria. This study is the first one which clarified the efficacy of IDPN in Japanese hemodialysis patients in RCT. The primary outcome studied was serum TTR level, and this did not change from study initiation to 12 weeks in either the intervention group or control group, nor was there a difference in the amount of change between the 2 groups. Most of the secondary outcomes, including nutritional laboratory tests (i.e., RBP, Tf, and Cu) and nutritional parameters (i.e., BMI, GNRI, SI, and NRI-JH) did not significantly change during the study or differ between the 2 groups. However, Zn and nPCR increased during the study in the intervention group and not in the control group. The food (energy and protein) intake did not decrease in the intervention group as it did in the control group, and increases in both energy and protein intake in the intervention group were different than the decreases observed in the control group. Finally, the amount of decrease in amino acid concentrations from before to after dialysis was less in the intervention group than in the control group, suggesting that IDPN may have mitigated some of the amino acid decrease that may ordinarily occur during dialysis.

Serum TTR is a sensitive mortality predictor for patients on hemodialysis [23], and increases in TTR have been associated with improvements in survival rates [10]. The findings in our study that serum TTR did not increase in the intervention group and that the amount of change in serum TTR did not differ between the intervention and control groups were likely related to an insufficient provision of nutrients or an inadequate administration period, or both. The IDPN solution used in this study, ENEFLUID_®_ 550 mL, contains 15 g amino acids and 310 kcal energy (including 37.5 g glucose and 10 g lipid), which should be sufficient to replace the 8 g to 12 g of amino acid losses that are known to occur during a single dialysis treatment [8]. However, the amounts of amino acids and energy provided in this study were smaller than those provided in some randomized clinical trials that have reported improvements in nutritional status using IDPN. In the study by Marsen, et al., IDPN containing 40.8 g amino acids and 820 kcal energy (including 81g glucose and 28.2 g lipid) was administered during dialysis 3 times a week for 4 months, and serum TTR increased [12]. In a study by Kittskulnam, et al., IDPN containing 50 g amino acids and 1100 kcal energy (including 125 g glucose and 38 g lipid) was administered during dialysis 3 times a week for 3 months, and although serum albumin increased, serum TTR did not change [13]. In addition, a systematic review of 12 clinical studies showed that IDPN improved nutritional parameters, including serum TTR and body weight, although it was not superior to ONS [24]. Importantly, for many of the studies included in that systematic review, the period of IDPN administration was longer than 6 months. Furthermore, compared to previous studies, the serum Alb levels of our study patients were lower and their CRP levels were higher than other studies’ patient [11–13], which may be one of the reasons why improvement with IDPN was not observed. Along with these other studies, our results suggest that IDPN containing amino acids, glucose, lipid, and a minimum of 800 kcal of energy may be required to reach the goal of nutritional status improvement within 3 to 4 months, and that IDPN using ENEFLUID_®_ may require a course of treatment of 6 months or more to confirm its utility in improving nutritional status, including serum TTR.

At 12 weeks, nPCR, commonly used to assess dietary protein intake in dialysis patients, increased in the intervention group but not in the control group, though these changes did not differ significantly between the 2 groups. On a related note, in the patient group which excluded the patients whose energy intake at the time of study initiation was less than a quarter or 2 times or more of the recommended amount, energy and protein intake increased in the intervention group and decreased in the control group; in particular, decrease of energy intake in the control group was statistically significant. Moreover, when the 2 groups were compared at 12 weeks, the changes in energy and protein intake were found to be different. We also performed an analysis using the larger study population which included all patients regardless of energy intake at the time of study initiation (**S3 Table**), and found that the changes in energy and protein intake were similar to the primary analysis results (**Table 5**). Others have reported that the intake of energy and protein in food has increased when IDPN has been used in patients receiving hemodialysis [11,13,25]. While our results are similar, they suggest that ENEFLUID_®_ may mitigate the decrease in food intake that might be expected in patients not receiving IDPN. In the study by Kittskulnam, et al., which also involved the administration of IDPN during hemodialysis for 3 months, patients in the intervention group exhibited increased energy intake, whereas those in the control group had decreased intake [13]. In addition, the levels of leptin, which exerts inhibitory effects on food intake, significantly increased in the control group, whereas they were unchanged in the intervention group. They postulated that a relative increase in food intake in the setting of IDPN may be the result of relief of anorexia due to the suppression of increases in leptin that would otherwise be occurring [13]. Although this is an attractive hypothesis, another recent study has demonstrated no association between serum leptin concentrations and anorexia in hemodialysis patients [26]. Leptin may be involved in improvements (relative to controls) in food intake that are associated with IDPN, but may not represent the entire explanation. Although one study referenced by Kittskulnam, et al., has shown that patients experienced a transient reduction in hyperleptinemia when an amino acid dialysate was used during peritoneal dialysis [27], Liu, et al., have reported that energy intake was actually higher in patients receiving IDPN containing only glucose than in those receiving IDPN containing both amino acids and glucose [11]. Thus, it remains unclear whether the relative increases in food intake we observed in patients receiving IDPN were due to reversing protein loss, addressing insufficient energy intake, or some other process. At least, we should measure serum leptin in the next study to clarify the association between leptin and IDPN. In our study, serum Zn concentrations increased in the intervention group but not the control group. Some have reported that in patients receiving hemodialysis, increases in serum Zn after oral Zn intake have been associated with decreases in serum leptin, a negative regulator of appetite control [28]. Others have reported that Zn supplementation has mitigated taste disorders [29,30]. It is possible that the relative increase in food intake experienced by patients receiving IDPN in our study might have been related to increased Zn concentrations and related improvements in taste and appetite. Moreover, providing supplemental nutrition through IDPN may have helped reverse PEW and mitigate the inflammatory processes, thereby stimulating appetite and increasing spontaneous oral intake. More detailed study on this point is needed in the future.

This is the first study to closely monitor the effect of IDPN on blood glucose during dialysis, on dialysis days, and on non-dialysis days, and to compare the results with those of the control group. Of particular interest, patients receiving IDPN in this study demonstrated low levels of hypoglycemia during dialysis. These results were similar to findings in our previous study [14] and suggest that the use of IDPN may help prevent hypoglycemia during dialysis. The risk for developing asymptomatic hypoglycemia has been reported to be highest within 24 hours of dialysis [31], and hypoglycemia increases the risk of death in patients with chronic kidney disease [32]. In addition, acute changes in blood glucose levels are associated with increases in oxidative stress and arteriosclerosis [33,34]. Thus, dialysis may be safer if techniques are utilized that prevent acute changes in blood glucose and asymptomatic hypoglycemia during and after dialysis. Based on our results, the use of ENEFLUID_®_ for IDPN may help improve the safety of hemodialysis.

No adverse events related to ENEFLUID_®_ were observed in this study. The study completion rate was only 85%, though this was similar to the completion rate in a similar study by Marsen, et al [12]. Elevations of C-reactive protein, a marker for inflammation, as well as T-Cho and triglyceride, both measures of lipid abnormality, were not observed in either study group. Also, BNP, a marker of excess body fluid, did increase in the control group but not in the intervention group over the course of the study. This suggests that the addition of the relatively small volume of administered ENEFLUID_®_, 550 mL, is not likely to result in excess body fluid.

When IDPN is performed, additional work is required of paramedical staff to prepare infusion solutions, operate infusion pumps, and connect infusion tubing to the dialysis circuit. The use of an all-in-one preparation made specifically for IDPN would facilitate the process of administration. ENEFLUID_®_ contains amino acids, glucose, lipid, minerals including trace elements, and water-soluble vitamins, all in a single bag. Although ENEFLUID_®_ is not made specifically for IDPN, it appears to be safe and its use has the potential to contribute to improved food intake, prevention of asymptomatic hypoglycemia, and suppression of plasma amino acid decreases during dialysis.

There are several limitations in this study. First, the sample size of 40 patients for this exploratory study was set based on what would be practical to achieve at each site. This sample size may have been too small to detect changes in the primary endpoint. Additionally, since this study is exploratory and no proper sample size calculation was provided, the results should be interpreted with caution. Second, the intervention period of only 12 weeks might have been too short to detect improvements in some of the nutritional parameters, including the primary endpoint. Third, this is an open-label study; therefore, the possibility that patient behavior, such as eating habits, or the medical staff’s examination practices may have been influenced cannot be ruled out. Fourth, not-measured nutritional components (e.g., vitamins and trace elements) might affect nutritional status. Finally, the study was limited to Japanese patients, and it is unknown whether the study results can be generalized to other races.

## Conclusions

In patients with mild to moderate malnutrition receiving ENEFLUID_®_ as IDPN for 12 weeks during maintenance hemodialysis, serum TTR, a mortality predictor for patients on hemodialysis, was not increased; however, decrease in protein intake was mitigated, and no adverse events were observed. A large-scale study involving at least 6 months of intervention is needed to more accurately determine the impact on nutritional status of ENEFLUID_®_ when used for IDPN.

## Supporting information

S1 Fig Examination schedule for evaluating outcomes over 12 weeks in 39 adult patients with malnutrition on maintenance hemodialysis (HD) beginning September through December 2022. (TIF)

S1 Table Nutritional Risk Index-Japanese Hemodialysis (NRI-JH) criteria used for study involving 39 patients with mild to moderate risk (5 to 10 points) malnutrition receiving maintenance hemodialysis. (DOCX)

S2 Table Composition of ENEFLUID_®_ 550 mL infusion used in study involving 39 patients with mild to moderate risk malnutrition receiving maintenance hemodialysis. (DOCX)

S3 Table Food intake results for 33 adult patients with malnutrition on maintenance hemodialysis, measured and compared at 2 timepoints, and compared between those receiving intradialytic parenteral nutrition (IDPN) and controls receiving no intervention, beginning September through December 2022. (DOCX)

S4 Table Laboratory test results for 39 adult patients with malnutrition on maintenance hemodialysis, measured and compared at 4 timepoints, and compared between those receiving intradialytic parenteral nutrition (IDPN) and controls receiving no intervention, beginning September through December 2022. (DOCX)

S5 Table Laboratory test results for glycemic control in 34 adult patients with malnutrition on maintenance hemodialysis (HD), measured and compared at 2 timepoints, and compared between those receiving intradialytic parenteral nutrition (IDPN) and controls receiving no intervention, beginning September through December 2022. (DOCX)
