# Supplementary figures and images for: Efficacy and safety of intradialytic parenteral nutrition using ENEFLUID® in malnourished patients receiving maintenance hemodialysis: An exploratory, multicenter, randomized, open-label study
Source: PLoS One. 2024 Dec 12;19(12):e0311671. doi: 10.1371/journal.pone.0311671 (PMC11637329; doi:10.1371/journal.pone.0311671)

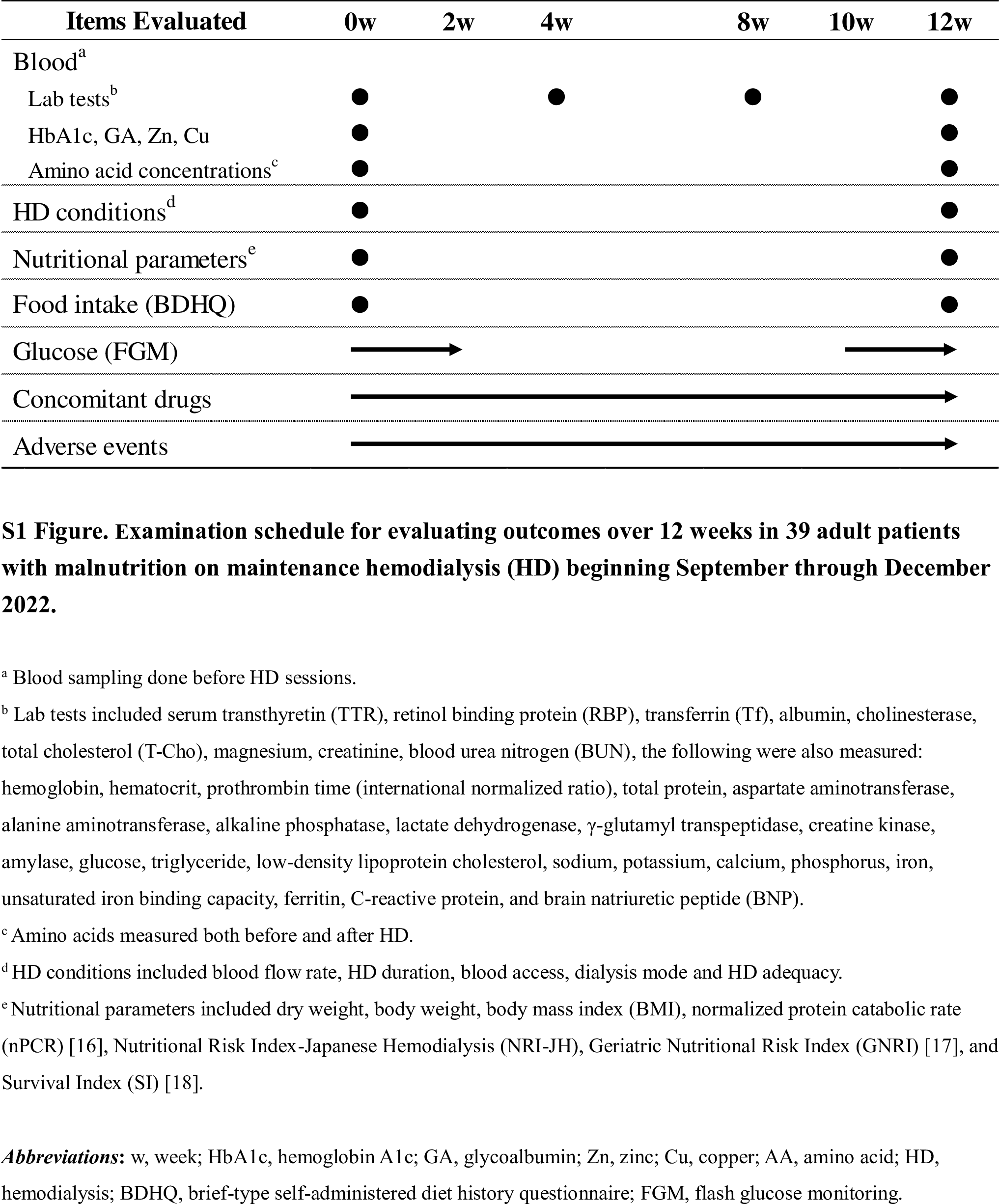

Supplement: S1 Fig — (TIF) [file pone.0311671.s001.tif]
