# Supplementary material for: Efficacy and safety of intradialytic parenteral nutrition using ENEFLUID® in malnourished patients receiving maintenance hemodialysis: An exploratory, multicenter, randomized, open-label study
Source: PLoS One. 2024 Dec 12;19(12):e0311671. doi: 10.1371/journal.pone.0311671 (PMC11637329; doi:10.1371/journal.pone.0311671)
Supplement: S1 Table — (DOCX) [file pone.0311671.s002.docx]

**S1 Table.** Nutritional Risk Index-Japanese Hemodialysis (NRI-JH) criteria^a^ used for study involving 39 patients with mild to moderate risk (5 to 10 points) malnutrition receiving maintenance hemodialysis

| **Parameter** | | **Criteria** | | **Score** |
| --- | --- | --- | --- | --- |
|  |  | Age < 65 years | Age ≥ 65 years |  |
| **Albumin**^b^, *g/dL* | | < 3.4 | < 3.2 | 4 |
| **Creatinine**, *mg/dL* | *Male* | < 11.6 | < 9.7 | 4 |
|  | *Female* | < 9.7 | < 8.0 |  |
| **Body mass index**, *kg/m^2^* | | < 20.0 | | 3 |
| **Total cholesterol**^c^, *mg/dL* | | < 130 | | 1 |

^a^ NRI-JH is the nutritional risk index used for predicting mortality in patients undergoing hemodialysis [15].

^b^ Levels determined using bromcresol purple method.

^c^ Serum total cholesterol not used in calculation of NRI-JH score in the study, because the study focused on malnutrition.
