# Supplementary material for: Efficacy and safety of intradialytic parenteral nutrition using ENEFLUID® in malnourished patients receiving maintenance hemodialysis: An exploratory, multicenter, randomized, open-label study
Source: PLoS One. 2024 Dec 12;19(12):e0311671. doi: 10.1371/journal.pone.0311671 (PMC11637329; doi:10.1371/journal.pone.0311671)
Supplement: S2 Table — (DOCX) [file pone.0311671.s003.docx]

**S2 Table.** Composition of ENEFLUID_®_ 550 mL infusion^a^ used in study involving 39 patients with mild to moderate risk malnutrition^b^ receiving maintenance hemodialysis

| **Ingredients** | | **Units** | **Values** |
| --- | --- | --- | --- |
| **Carbohydrates** | |  |  |
|  | Dextrose | *g* | 37.5 |
| **Amino acids** | |  |  |
|  | Total free amino acids | *g* | 15 |
|  | Total nitrogen | *g* | 2.37 |
|  | Essential/nonessential amino acids | - | 1.8 |
| **Fat (Lipid)** | |  |  |
|  | Purified soybean oil | *g* | 10 |
| **Electrolytes** | |  |  |
|  | Na^+^ | *mEq* | 17.5 |
|  | K^+^ | *mEq* | 10 |
|  | Mg^2+^ | *mEq* | 2.5 |
|  | Ca^2+^ | *mEq* | 2.5 |
|  | Cl^-^ | *mEq* | 17.5 |
|  | SO_4_^2-^ | *mEq* | 2.5 |
|  | Acetate^-^ | *mEq* | 8.2 |
|  | Gluconate^-^ | *mEq* | 2.5 |
|  | L-Lactate^-^ | *mEq* | 10.5 |
|  | Citrate^3−^ | *mEq* | 3.2 |
|  | P | *mmol* | 5 |
|  | Zn | *μmol* | 2.5 |
| **Vitamins** | |  |  |
|  | Thiamine chloride hydrochloride | *mg* | 1.91 |
|  | Riboflavin sodium phosphate | *mg* | 1.15 |
|  | Pyridoxine hydrochloride | *mg* | 1.83 |
|  | Cyanocobalamin | *μg* | 1.25 |
|  | Nicotinamide | *mg* | 10 |
|  | Panthenol | *mg* | 3.52 |
|  | Folic acid | *μg* | 150 |
|  | Biotin | *μg* | 15 |
|  | Ascorbic acid | *mg* | 50 |
| **pH** | | | Approx. 6.4 |
| **Osmotic pressure ratio (relative to saline solution)** | | | Approx. 3 |
| **Total calories** | | *kcal* | 310 |
| **Non-protein calories** | | *kcal* | 250 |

^a^ ENEFLUID_®_ (Otsuka Pharmaceutical Factory, Inc.; Tokushima, Japan) as intradialytic parenteral nutrition (IDPN).

^b^ Patients in the study had scores ranging from 5 to 10 points, based on the Nutritional Risk Index-Japanese Hemodialysis (NRI-JH) criteria.
