# Supplementary material for: Efficacy and safety of intradialytic parenteral nutrition using ENEFLUID® in malnourished patients receiving maintenance hemodialysis: An exploratory, multicenter, randomized, open-label study
Source: PLoS One. 2024 Dec 12;19(12):e0311671. doi: 10.1371/journal.pone.0311671 (PMC11637329; doi:10.1371/journal.pone.0311671)
Supplement: S3 Table — (DOCX) [file pone.0311671.s004.docx]

**S3 Table**. Food intake^a^ results for 33 adult patients with malnutrition on maintenance hemodialysis, measured and compared at 2 timepoints, and compared between those receiving intradialytic parenteral nutrition (IDPN) and controls receiving no intervention, beginning September through December 2022

|  | **Intervention (IDPN) group** | | | **Control group** | | | | **Difference (Intervention - Control)** | | |  |
| --- | --- | --- | --- | --- | --- | --- | --- | --- | --- | --- | --- |
|  | n=15 | | | n=18 | | | |  |  |  |  |
|  | mean (95% CI) | P-value^b^ | | mean (95% CI) | | P-value^b^ | | mean (95% CI) | P-value^c^ | |  |
| **Energy intake**, *kcal/kg/day* | | | | | | | | | | | |
| Study initiation day | 23.3 (18.5 to 28.2) | | – | | 33.4 (26.2 to 40.6) | | – | – | | – |  |
| 12 weeks | 25.5 (20.8 to 30.3) | | – | | 28.6 (22.3 to 34.9) | | – | – | | – |  |
| Change at 12 weeks | 2.2 (-1.8 to 6.2) | | 0.25 | | -4.8 (-8.8 to -0.8) | | **0.02** | 7.0 (1.5 to 12.5) | | **0.01** |  |
| **Protein intake**, *g/kg/day* | | | | | | | | | | | |
| Study initiation day | 0.85 (0.61 to 1.09) | | – | | 1.51 (0.99 to 2.03) | | – | – | | – |  |
| 12 weeks | 0.99 (0.73 to 1.25) | | – | | 1.25 (0.87 to 1.63) | | – | – | | – |  |
| Change at 12 weeks | 0.14 (-0.01 to 0.28) | | 0.06 | | -0.26 (-0.57 to 0.04) | | 0.08 | 0.40 (0.06 to 0.74) | | **0.02** |  |
| **Animal protein intake**, *g/kg/day* | | | | | | | | | | | |
| Study initiation day | 0.50 (0.33 to 0.67) | | – | | 0.99 (0.53 to 1.44) | | – | – | | – |  |
| 12 weeks | 0.59 (0.41 to 0.78) | | – | | 0.79 (0.49 to 1.08) | | – | – | | – |  |
| Change at 12 weeks | 0.10 (-0.02 to 0.21) | | 0.10 | | -0.20 (-0.49 to 0.09) | | 0.16 | 0.30 (-0.03 to 0.62) | | 0.07 |  |
| **Plant protein intake**, *g/kg/day* | | | | | | | | | | | |
| Study initiation day | 0.35 (0.27 to 0.43) | | – | | 0.53 (0.42 to 0.63) | | – | – | | – |  |
| 12 weeks | 0.39 (0.31 to 0.48) | | – | | 0.46 (0.37 to 0.56) | | – | – | | – |  |
| Change at 12 weeks | 0.04 (-0.01 to 0.10) | | 0.11 | | -0.06 (-0.11 to -0.01) | | **0.03** | 0.10 (0.03 to 0.18) | | **0.007** |  |

^a^ Data obtained using the brief-type self-administered diet history questionnaire (BDHQ) [19].

^b^ 12 weeks vs. study initiation day, P-values based on paired-samples t-test.

^c^ Intervention group vs Control group, P-values based on unpaired t-test.

***Abbreviation:*** CI, confidence interval.
