# Supplementary material for: Efficacy and safety of intradialytic parenteral nutrition using ENEFLUID® in malnourished patients receiving maintenance hemodialysis: An exploratory, multicenter, randomized, open-label study
Source: PLoS One. 2024 Dec 12;19(12):e0311671. doi: 10.1371/journal.pone.0311671 (PMC11637329; doi:10.1371/journal.pone.0311671)
Supplement: S4 Table — (DOCX) [file pone.0311671.s005.docx]

**S4 Table**. Laboratory test results for 39 adult patients with malnutrition on maintenance hemodialysis, measured and compared at 4 timepoints, and compared between those receiving intradialytic parenteral nutrition (IDPN) and controls receiving no intervention, beginning September through December 2022

|  | **Intervention group** | | **Control group** | | | **Difference (Intervention - Control)** | | |
| --- | --- | --- | --- | --- | --- | --- | --- | --- |
|  | n = 20 | | n = 19 | | |  |  |  |
|  | LS mean (95% CI) | P-value^a^ | LS mean (95% CI) | P-value^a^ | | LS mean (95% CI) | | P-value^b^ |
| **Hemoglobin**, *g/dL* | | | | | | | | |
| Study initiation day | 10.8 (10.3 to 11.3) | – | 11.0 (10.5 to 11.5) | | – | – | – | |
| 4 weeks | 11.1 (10.6 to 11.6) | – | 11.0 (10.5 to 11.5) | | – | – | – | |
| 8 weeks | 10.4 (9.9 to 10.9) | – | 10.8 (10.2 to 11.3) | | – | – | – | |
| 12 weeks | 10.7 (10.2 to 11.3) | – | 10.8 (10.3 to 11.3) | | – | – | – | |
| Change at 12 weeks | -0.1 (-0.7 to 0.5) | 0.77 | -0.2 (-0.7 to 0.4) | | 0.55 | 0.1 (-0.7 to 0.9) | 0.84 | |
| **Hematocrit**, *%* | | | | | | | | |
| Study initiation day | 33.7 (32.1 to 35.4) | – | 33.9 (32.3 to 35.5) | | – | – | – | |
| 4 weeks | 34.7 (33.2 to 36.3) | – | 34.2 (32.6 to 35.8) | | – | – | – | |
| 8 weeks | 32.8 (31.2 to 34.4) | – | 33.6 (31.9 to 35.2) | | – | – | – | |
| 12 weeks | 33.3 (31.7 to 35.0) | – | 33.6 (32.0 to 35.2) | | – | – | – | |
| Change at 12 weeks | -0.4 (-2.3 to 1.4) | 0.66 | -0.3 (-2.1 to 1.5) | | 0.75 | -0.1 (-2.7 to 2.5) | 0.92 | |
| **Prothrombin time (international normalized ratio)** | | | | | | | | |
| Study initiation day | 1.16 (0.76 to 1.56) | – | 1.31 (0.92 to 1.69) | | – | – | – | |
| 4 weeks | 1.16 (0.77 to 1.55) | – | 1.29 (0.91 to 1.67) | | – | – | – | |
| 8 weeks | 1.17 (0.77 to 1.56) | – | 1.33 (0.94 to 1.72) | | – | – | – | |
| 12 weeks | 1.18 (0.75 to 1.61) | – | 1.65 (1.25 to 2.04) | | – | – | – | |
| Change at 12 weeks | 0.02 (-0.47 to 0.50) | 0.95 | 0.34 (-0.09 to 0.77) | | 0.12 | -0.33 (-0.97 to 0.32) | 0.32 | |
| **Total protein**, *g/dL* | | | | | | | | |
| Study initiation day | 6.2 (6.0 to 6.4) | – | 6.3 (6.1 to 6.5) | | – | – | – | |
| 4 weeks | 6.4 (6.2 to 6.6) | – | 6.3 (6.1 to 6.5) | | – | – | – | |
| 8 weeks | 6.3 (6.1 to 6.5) | – | 6.3 (6.1 to 6.5) | | – | – | – | |
| 12 weeks | 6.3 (6.1 to 6.5) | – | 6.4 (6.1 to 6.6) | | – | – | – | |
| Change at 12 weeks | 0.1 (-0.1 to 0.3) | 0.29 | 0.1 (-0.1 to 0.3) | | 0.36 | 0.0 (-0.3 to 0.3) | 0.91 | |
| **Asparate aminotransferase**, *U/L* | | | | | | | | |
| Study initiation day | 17 (13 to 21) | – | 14 (10 to 18) | | – | – | – | |
| 4 weeks | 19 (15 to 23) | – | 16 (12 to 20) | | – | – | – | |
| 8 weeks | 17 (13 to 21) | – | 14 (10 to 18) | | – | – | – | |
| 12 weeks | 20 (16 to 24) | – | 14 (10 to 18) | | – | – | – | |
| Change at 12 weeks | 3 (-1 to 7) | 0.13 | 0 (-4 to 4) | | 0.99 | 3 (-3 to 9) | 0.27 | |
| **Alanine aminotransferase**, *U/L* | | | | | | | | |
| Study initiation day | 11 (8 to 13) | – | 9 (7 to 12) | | – | – | – | |
| 4 weeks | 12 (9 to 14) | – | 11 (8 to 14) | | – | – | – | |
| 8 weeks | 11 (8 to 14) | – | 10 (7 to 13) | | – | – | – | |
| 12 weeks | 13 (10 to 16) | – | 9 (6 to 12) | | – | – | – | |
| Change at 12 weeks | 2 (-1 to 6) | 0.18 | -1 (-4 to 3) | | 0.72 | 3 (-2 to 8) | 0.23 | |
| **Alkaline phosphatase**, *U/L* | | | | | | | | |
| Study initiation day | 87 (73 to 101) | – | 72 (57 to 86) | | – | – | – | |
| 4 weeks | 92 (78 to 106) | – | 73 (58 to 87) | | – | – | – | |
| 8 weeks | 98 (84 to 113) | – | 75 (60 to 89) | | – | – | – | |
| 12 weeks | 92 (78 to 107) | – | 77 (63 to 92) | | – | – | – | |
| Change at 12 weeks | 6 (-7 to 18) | 0.38 | 5 (-7 to 18) | | 0.41 | 0.0 (-17 to 18) | 0.97 | |
| **Lactate dehydrogenase**, *U/L* | | | | | | | | |
| Study initiation day | 192 (169 to 215) | – | 208 (184 to 231) | | – | – | – | |
| 4 weeks | 207 (184 to 230) | – | 219 (195 to 242) | | – | – | – | |
| 8 weeks | 187 (164 to 210) | – | 211 (187 to 235) | | – | – | – | |
| 12 weeks | 188 (164 to 212) | – | 210 (187to 234) | | – | – | – | |
| Change at 12 weeks | -4 (-25 to 16) | 0.68 | 3 (-18 to 23) | | 0.79 | -7 (-36 to 22) | 0.63 | |
| **γ-Glutamyl transpeptidase**, *U/L* | | | | | | | | |
| Study initiation day | 24 (17 to 31) | – | 18 (11 to 25) | | – | – | – | |
| 4 weeks | 24 (17 to 30) | – | 19 (12 to 26) | | – | – | – | |
| 8 weeks | 25 (18 to 32) | – | 20 (13 to 27) | | – | – | – | |
| 12 weeks | 27 (20 to 34) | – | 20 (13 to 27) | | – | – | – | |
| Change at 12 weeks | 3 (-3 to 8) | 0.33 | 2 (-4 to 8) | | 0.50 | 1 (-7 to 9) | 0.83 | |
| **Creatine kinase**, *U/L* | | | | | | | | |
| Study initiation day | 65 (40 to 90) | – | 92 (66 to 118) | | – | – | – | |
| 4 weeks | 66 (40 to 92) | – | 109 (83 to 136) | | – | – | – | |
| 8 weeks | 63 (37 to 90) | – | 85 (58 to 113) | | – | – | – | |
| 12 weeks | 66 (39 to 94) | – | 93 (65 to 121) | | – | – | – | |
| Change at 12 weeks | 1 (-29 to 31) | 0.93 | 1 (-30 to 31) | | 0.97 | 1 (-42 to 43) | 0.97 | |
| **Amylase**, *U/L* | | | | | | | | |
| Study initiation day | 117 (86 to 148) | – | 134 (105 to 163) | | – | – | – | |
| 4 weeks | 124 (94 to 155) | – | 145 (116 to 175) | | – | – | – | |
| 8 weeks | 125 (94 to 156) | – | 136 (107 to 165) | | – | – | – | |
| 12 weeks | 121 (91 to 152) | – | 130 (101 to 159) | | – | – | – | |
| Change at 12 weeks | 4 (-26 to 34) | 0.77 | -4 (-33 to 24) | | 0.77 | 9 (-33 to 50) | 0.68 | |
| **Glucose**, *mg/dL* | | | | | | | | |
| Study initiation day | 120 (100 to 140) | – | 149 (129 to 170) | | – | – | – | |
| 4 weeks | 129 (109 to 148) | – | 150 (130 to 171) | | – | – | – | |
| 8 weeks | 110 (90 to 130) | – | 142 (121 to 163) | | – | – | – | |
| 12 weeks | 134 (113 to 154) | – | 130 (109 to 151) | | – | – | – | |
| Change at 12 weeks | 14 (-8 to 36) | 0.21 | -20 (-41 to 2) | | **0.08** | 33 (3 to 64) | **0.03** | |
| **Triglyceride**, *mg/dL* | | | | | | | | |
| Study initiation day | 78 (62 to 94) | – | 75 (58 to 91) | | – | – | – | |
| 4 weeks | 87 (71 to 103) | – | 79 (62 to 96) | | – | – | – | |
| 8 weeks | 89 (72 to 105) | – | 78 (61 to 95) | | – | – | – | |
| 12 weeks | 83 (66 to 101) | – | 77 (60 to 94) | | – | – | – | |
| Change at 12 weeks | 5 (-17 to 27) | 0.65 | 2 (-20 to 24) | | 0.84 | 3 (-28 to 34) | 0.86 | |
| **Low-density lipoprotein cholesterol**, *mg/dL* | | | | | | | | |
| Study initiation day | 74 (61 to 87) | – | 69 (56 to 83) | | – | – | – | |
| 4 weeks | 76 (62 to 89) | – | 61 (47 to 75) | | – | – | – | |
| 8 weeks | 77 (63 to 90) | – | 61 (47 to 75) | | – | – | – | |
| 12 weeks | 70 (56 to 84) | – | 61 (47 to 75) | | – | – | – | |
| Change at 12 weeks | -4 (-19 to 11) | 0.61 | -9 (-23 to 6) | | 0.26 | 5 (-16 to 26) | 0.66 | |
| **Sodium**, *mEq/L* | | | | | | | | |
| Study initiation day | 139 (138 to 140) | – | 138 (137 to 140) | | – | – | – | |
| 4 weeks | 139 (138 to 141) | – | 138 (137 to 140) | | – | – | – | |
| 8 weeks | 139 (137 to 140) | – | 138 (137 to 140) | | – | – | – | |
| 12 weeks | 139 (138 to 141) | – | 138 (137 to 140) | | – | – | – | |
| Change at 12 weeks | 0 (-2 to 2) | 0.83 | 0 (-2 to 2) | | 0.89 | 0 (-2 to 3) | 0.96 | |
| **Potassium**, *mEq/L* | | | | | | | | |
| Study initiation day | 4.6 (4.3 to 5.0) | – | 4.7 (4.3 to 5.1) | | – | – | – | |
| 4 weeks | 5.1 (4.7 to 5.4) | – | 4.5 (4.1 to 4.9) | | – | – | – | |
| 8 weeks | 5.0 (4.7 to 5.4) | – | 4.7 (4.3 to 5.0) | | – | – | – | |
| 12 weeks | 4.8 (4.4 to 5.1) | – | 4.8 (4.4 to 5.2) | | – | – | – | |
| Change at 12 weeks | 0.1 (-0.3 to 0.5) | 0.57 | 0.1 (-0.3 to 0.5) | | 0.65 | 0.0 (-0.5 to 0.5) | 0.94 | |
| **Calcium**, *mg/dL* | | | | | | | | |
| Study initiation day | 8.3 (8.0 to 8.6) | – | 8.6 (8.2 to 8.9) | | – | – | – | |
| 4 weeks | 8.4 (8.1 to 8.7) | – | 8.6 (8.3 to 9.0) | | – | – | – | |
| 8 weeks | 8.3 (8.0 to 8.6) | – | 8.5 (8.2 to 8.8) | | – | – | – | |
| 12 weeks | 8.5 (8.2 to 8.9) | – | 8.4 (8.1 to 8.8) | | – | – | – | |
| Change at 12 weeks | 0.2 (-0.1 to 0.6) | 0.24 | -0.1 (-0.5 to 0.2) | | 0.48 | 0.3 (-0.2 to 0.8) | 0.18 | |
| **Magnesium**, *mg/dL* | | | | | | | | |
| Study initiation day | 2.5 (2.3 to 2.7) | – | 2.4 (2.3 to 2.6) | | – | – | – | |
| 4 weeks | 2.6 (2.4 to 2.8) | – | 2.5 (2.3 to 2.6) | | – | – | – | |
| 8 weeks | 2.6 (2.4 to 2.7) | – | 2.5 (2.4 to 2.7) | | – | – | – | |
| 12 weeks | 2.5 (2.4 to 2.7) | – | 2.4 (2.3 to 2.6) | | – | – | – | |
| Change at 12 weeks | 0.0 (-0.1 to 0.2) | 0.55 | 0.0 (-0.1 to 0.2) | | 0.75 | 0.0 (-0.2 to 0.2) | 0.84 | |
| **Phosphorus**, *mg/dL* | | | | | | | | |
| Study initiation day | 4.8 (4.2 to 5.3) | – | 5.0 (4.5 to 5.6) | | – | – | – | |
| 4 weeks | 5.5 (5.0 to 6.0) | – | 4.7 (4.2 to 5.2) | | – | – | – | |
| 8 weeks | 5.7 (5.1 to 6.2) | – | 4.6 (4.1 to 5.2) | | – | – | – | |
| 12 weeks | 5.2 (4.7 to 5.8) | – | 4.8 (4.3 to 5.3) | | – | – | – | |
| Change at 12 weeks | 0.5 (-0.2 to 1.1) | 0.14 | -0.2 (-0.9 to 0.4) | | 0.45 | 0.7 (-0.2 to 1.6) | 0.12 | |
| **Iron**, *μg/dL* | | | | | | | | |
| Study initiation day | 55 (43 to 68) | – | 56 (43 to 69) | | – | – | – | |
| 4 weeks | 68 (55 to 81) | – | 57 (44 to 70) | | – | – | – | |
| 8 weeks | 59 (46 to 71) | – | 52 (39 to 65) | | – | – | – | |
| 12 weeks | 61 (47 to 74) | – | 63 (50 to 77) | | – | – | – | |
| Change at 12 weeks | 6 (-10 to 21) | 0.48 | 7 (-9 to 23) | | 0.38 | -1 (-24 to 21) | 0.90 | |
| **Unsaturated iron binding capacity**, *μg/dL* | | | | | | | | |
| Study initiation day | 183 (159 to 208) | – | 176 (150 to 201) | | – | – | – | |
| 4 weeks | 182 (157 to 206) | – | 184 (159 to 209) | | – | – | – | |
| 8 weeks | 180 (156 to 205) | – | 186 (160 to 211) | | – | – | – | |
| 12 weeks | 173 (147 to 199) | – | 173 (147 to 199) | | – | – | – | |
| Change at 12 weeks | -10 (-34 to 13) | 0.38 | -3 (-26 to 21) | | 0.83 | -8 (-41 to 25) | 0.64 | |
| **Ferritin**, *ng/mL* | | | | | | | | |
| Study initiation day | 88.0 (55.5 to 120.5) | – | 76.8 (43.4 to 110.1) | | – | – | – | |
| 4 weeks | 82.8 (49.9 to 115.7) | – | 72.5 (38.7 to 106.3) | | – | – | – | |
| 8 weeks | 108.3 (75.5 to 141.0) | – | 78.6 (45.0 to 112.2) | | – | – | – | |
| 12 weeks | 111.4 (77.4 to 145.3) | – | 91.9 (58.2 to 125.7) | | – | – | – | |
| Change at 12 weeks | 23.4 (-6.2 to 52.9) | 0.12 | 15.2 (-13.8 to 44.1) | | 0.30 | 8.2 (-33.1 to 49.5) | 0.70 | |
| **C-reactive protein**, *mg/dL* | | | | | | | | |
| Study initiation day | 1.41 (0.34 to 2.48) | – | 0.63 (0.00 to 1.73) | | – | – | – | |
| 4 weeks | 1.19 (0.12 to 2.26) | – | 0.28 (0.00 to 1.37) | | – | – | – | |
| 8 weeks | 1.70 (0.63 to 2.78) | – | 0.35 (0.00 to 1.45) | | – | – | – | |
| 12 weeks | 1.55 (0.44 to 2.67) | – | 0.69 (0.00 to 1.80) | | – | – | – | |
| Change at 12 weeks | 0.14 (-0.93 to 1.21) | 0.79 | 0.06 (-1.01 to 1.12) | | 0.92 | 0.09 (-1.42 to 1.60) | 0.91 | |
| **Brain natriuretic peptide**, *pg/mL* | | | | | | | | |
| Study initiation day | 645 (-244 to 1534) | – | 1216 (301 to 2130) | | – | – | – | |
| 4 weeks | 599 (-292 to 1490) | – | 1371 (453 to 2288) | | – | – | – | |
| 8 weeks | 470 (-423 to 1362) | – | 1468 (550. to 2387) | | – | – | – | |
| 12 weeks | 486 (-409 to 1381) | – | 1641 (724 to 2557) | | – | – | – | |
| Change at 12 weeks | -159 (-491 to 173) | 0.34 | 425 (95 to 754) | | **0.01** | -584 (-1051 to -116) | **0.02** | |

^a^ 12 weeks vs. study initiation day, P-values based on mixed-effects model for repeated measures, and a first-order autoregressive covariance structure was used.

^b^ Intervention group vs Control group, P-values based on mixed-effects model for repeated measures, and a first-order autoregressive covariance structure was used.

***Abbreviations***: LS, least squares; CI, confidence interval.
