# Supplementary material for: Efficacy and safety of intradialytic parenteral nutrition using ENEFLUID® in malnourished patients receiving maintenance hemodialysis: An exploratory, multicenter, randomized, open-label study
Source: PLoS One. 2024 Dec 12;19(12):e0311671. doi: 10.1371/journal.pone.0311671 (PMC11637329; doi:10.1371/journal.pone.0311671)
Supplement: S5 Table — (DOCX) [file pone.0311671.s006.docx]

**S5 Table.** Laboratory test results for glycemic control in 34 adult patients with malnutrition on maintenance hemodialysis (HD), measured and compared at 2 timepoints, and compared between those receiving intradialytic parenteral nutrition (IDPN) and controls receiving no intervention, beginning September through December 2022

|  | **Intervention (IDPN) group** | | | **Control group** | | | **Difference (Intervention - Control)** | |
| --- | --- | --- | --- | --- | --- | --- | --- | --- |
|  | n | mean (95% CI) | P-value^a^ | n | mean (95% CI) | P-value^a^ | mean (95% CI) | P-value^b^ |
| **HemoglobinA1c**, *%* | | | | | | | | |
| Study initiation day | 16 | 5.2 (4.9 to 5.5) | – | 18 | 5.6 (5.1 to 6.0) | – | – | – |
| 12 weeks |  | 5.3 (5.0 to 5.6) | – |  | 5.5 (5.1 to 6.0) | – | – | – |
| Change at 12 weeks |  | 0.1 (0.0 to 0.3) | 0.12 |  | -0.1 (-0.2 to 0.1) | 0.44 | 0.2 (0.0 to 0.4) | 0.08 |
| **Glycoalbumin**, *%* | | | | | | | | |
| Study initiation day | 16 | 17.4 (15.9 to 18.8) | – | 18 | 18.7 (16.3 to 21.1) | – | – | – |
| 12 weeks |  | 17.5 (15.8 to 19.3) | – |  | 18.9 (16.7 to 21.2) | – | – | – |
| Change at 12 weeks |  | 0.2 (-0.5 to 0.9) | 0.58 |  | 0.2 (-0.3 to 0.7) | 0.37 | 0.0 (-0.8 to 0.8) | 0.93 |

^a^ 12 weeks vs. study initiation day, P-values based on paired-samples t-test.

^b^ Intervention group vs Control group, P-values based on unpaired t-test.

***Abbreviation:*** CI, confidence interval.
